# Selinexor, a selective inhibitor of nuclear export (SINE), shows enhanced activity in combination with PD-1/PD-L1 blockade in syngeneic murine models of colon cancer and melanoma

**DOI:** 10.1186/2051-1426-3-S2-P355

**Published:** 2015-11-04

**Authors:** Matthew R Farren, Reena Shakya, Rebecca Hennessey, Thomas Mace, Jennifer Yang, Omar Elnaggar, Gregory Young, Yosef Landesman, Robert Carlson, Sivan Elloul, Marsha Crochiere, Christin Burd, Gregory Lesinski

**Affiliations:** 1 Department of Internal Medicine, James Comprehensive Cancer Center, The Ohio State University, Columbus, OH, USA; 2 The Ohio State University, Columbus, OH, USA; 3 Department of Molecular Genetics, James Comprehensive Cancer Center, The Ohio State University, Columbus, OH, USA; 4 Department of Internal Medicine, James Comprehensive Cancer Center, The Ohio State University, Columbus, OH, USA; 5 Center for Biostatistics, The Ohio State University, Columbus, OH, USA; 6 Karyopharm Therapeutics, Newton, MA, USA

## 

Exportin-1 (XPO1) is a nuclear export protein with >220 cargo proteins, including tumor suppressors and cell cycle modulators. Selinexor is a SINE (Selective Inhibitor of Nuclear Export) compound that has been administered to >900 cancer patients in Phase I and II trials to date, with evidence of efficacy and tolerability. Selinexor blocks nuclear export of NFAT1c, STAT1 and STAT3, which are implicated in regulating the inhibitory T cell receptor PD-1 and its ligand, PD-L1. We hypothesized that selinexor would upregulate T cell checkpoint molecule expression, and that combination treatment with anti-PD-1 or anti-PD-L1 would thereby enhance the ability of selinexor to elicit antitumor activity.

Selinexor increased PD-1 gene expression by ~2-fold in normal lymphocytes and induced PD-L1 gene expression in tumor cell lines. Mice bearing syngeneic colon tumors (colon26) treated with selinexor and anti-PD-1 for 2 weeks demonstrated a significant reduction in tumor growth rate (P < 0.05), while monotherapy with either agent had no significant effect on tumor growth. Similar results were obtained in mice bearing syngeneic B16F10 melanoma tumors, whereby combined treatment with selinexor + anti-PD-1 was superior to either single agent alone (p < 0.034). Combined therapy of mice bearing B16F10 tumors with selinexor and anti-PD-L1 was similarly effective, with significantly smaller tumors at the study endpoint (p < 0.001). No weight loss or signs of toxicity were evident in any *in vivo* study.

Immunophenotypic analysis by flow cytometry revealed that selinexor alone or in combination with anti-PD-1/anti-PD-L1 significantly increased the percentage of splenic NK cells (p≤0.050), while selinexor ± anti-PD-L1 significantly increased the percentage of splenic Th1 T cells (p≤0.011), all compared to vehicle treated mice. Interestingly, combining selinexor with anti-PD-L1 significantly decreased the percentage of splenocytes that expressed PD-L1 (p < 0.001). These data indicate that the efficacy of selinexor may be enhanced by disrupting the pre-existing PD-1/PD-L1 signaling in effector cells (T and NK cells).

Altogether, these data suggest that the efficacy of selinexor in combination with anti-PD-1 or anti PD L1 in mouse syngeneic tumor models may be due to both disrupting immunosuppressive PD-1/PD-L1 signaling and increasing the frequency of potentially tumor reactive NK cells and Th1 T cells. This provides a rational basis for this treatment combination as a novel therapeutic approach for advanced cancer.

